# The Impact of* IL-16* 3′UTR Polymorphism rs859 on Lung Carcinoma Susceptibility among Chinese Han Individuals

**DOI:** 10.1155/2018/8305745

**Published:** 2018-12-24

**Authors:** Manyun Zhuo, Xiaohong Zhuang, Wenjun Tang, Junnv Xu, Chengsheng Zhang, Xiaoying Qian, Jie Wang, Hui Liu, Yueli Liu, Zhong Chen, Haifeng Lin

**Affiliations:** ^1^Department of Medical Oncology, The Second Affiliated Hospital of Hainan Medical University, Haikou, Hainan 570311, China; ^2^Department of Nursing, The Second Affiliated Hospital of Hainan Medical University, Haikou, Hainan 570311, China; ^3^Department of Anatomy, Hainan Medical University, Haikou, Hainan 571199, China; ^4^Department of Pharmacology, Hainan Medical University, Haikou, Hainan 571199, China; ^5^Hainan Provincial Third People's Hospital, Sanya, Hainan 572000, China

## Abstract

Lung carcinoma is the most common cancer and cause of cancer deaths among both males and females in China. Previously, genetic variants located in gene untranslated region have been well established as interfering factors in mRNA translation and confirmed playing critical roles in lung oncogenesis. However, the correlation between polymorphisms in gene 3′ untranslated region and lung cancer risk is less reported in China Han population. In this study, polymorphisms in 3′-untranslated region of* IL-16*,* CYP24A1*, and* FBN1* were determined in 322 lung cancer patients and 384 healthy controls with the usage of Sequenom MassARRAY. The correlation between selected variants and lung cancer risk was examined by unconditional logistic regression analysis with or without adjustments for age, gender, smoking status, and alcohol drinking status. Additionally, stratification analysis was applied to detect the associations of SNPs with lung cancer in different subgroups. As the results, significant relationships were found between* IL-16* rs859 and lung cancer susceptibility in recessive model (OR= 0.65, 95% CI: 0.44-0.96,* P*= 0.029) and log-additive model (OR= 0.76, 95% CI: 0.60-0.96,* P*= 0.019). Moreover, adjusted stratified analysis also revealed the important effects of* IL-16* rs859 on lung cancer risk among individuals aged older than 50, males, and nondrinkers.* IL-16* rs859 showed statistically significant evidence associated with susceptibility to lung adenocarcinoma and lung small cell carcinoma in Chinese Han population as well. Our research demonstrated that genetic variant rs859 of* IL-16* 3′UTR was associated with lung cancer risk in Chinese Han population and the result might be exploited as a new biomarker for lung cancer assessment and prevention.

## 1. Introduction

Lung cancer is one of the leading causes of cancer-related death worldwide and the most frequent and aggressive malignancies in China [1]. With a relatively high morbidity and mortality, lung cancer has become a great threat among males and females in both more and less developed areas [2]. It has been statistical confirmed that non-small-cell lung cancer (NSCLC) cases account for approximately 85% of all lung cancers and the five-year overall survival rate of the patients is lower than 20% [3, 4]. Moreover, a considerable number of patients are identified suffering from an advanced stage of disease at time of diagnosis, which implies the poor prognosis and high recurrence rate during the treatment [3]. While significant improvements have been implemented on surgery, radiotherapy, and chemotherapy, limitations still exist in terms of application of these therapeutic methods due to the different and complicated individual conditions.

Currently, numerous studies have underscored multiple factors that could contribute to the lung tumorigenesis. Convincing evidence indicates the causal roles of air pollutants in the increased risk of lung cancer [5]. A meta-analysis showed statistically significant correlation between long-term exposures to smoky coal, environmental tobacco smoke (ETS), and elevated lung cancer incidence in all involved groups from China [6]. Smoking is another leading reason which a large amount of lung cancer cases could be attributable to [7, 8]. Nevertheless, there are still approximately 10% to 25% of lung cancer cases could not be ascribed to smoking and a great majority of them have been validated associated with internal genetic mutations and abnormal regulation [9]. Recent studies focusing on lung cancer causes in never-smokers have discovered that these patients possess characteristics distinct from those in smokers [10]. Additionally, drug effects might be subject to polygenic determinants as well. Therefore, molecular and genetic research could not only provide the novel elucidation of potential mechanism in lung carcinogenesis, but also explore new therapeutic targets for better treatment for lung cancer.

Untranslated region (UTR) at 3′ end of the mRNAs is well established playing a pivotal role in translational regulation, subcellular localization, and stability maintenance [11]. In mammals, 3′ untranslated regions (3′UTR) of mRNAs contain conserved 6-8mer sequences that match the seed regions of corresponding miRNAs. MiRNAs generally serve as negative regulators of gene expression through sequence-specific complementarity and finally resulting in mRNAs cleavage and repression via guiding associated effector complexes to the mRNAs [12]. Moreover, the RNA-localization elements could also be detected in the 3′ UTR of mRNAs, coding the message directing the mRNA-protein complex towards their specific subcellular destinations [11].* Cis*-elements in 3′UTR have also been proven involved in mRNA degradation processes and further influence the stability and abundance of mRNAs [13]. Previous work has uncovered the independent roles of SNPs distributed in the 3′ UTRs of genes. SNPs located in the 3′UTR targeting sequences are able to influence the regulatory effects of miRNAs, which might lead to disorder of gene expression in tumorigenesis. Accumulating studies have demonstrated the significant polymorphic variants resided on miRNA-targeting seed regions which contribute to unnatural interactions and cancer susceptibility [14–16]. However, because of the longer sequence and high complexity of mRNA 3′UTR in human as well as its multiple functions, variants located in other positions should be attached more importance [17]. These polymorphisms within the 3′UTR exert functional roles in gene expression regulation during the complex process of oncogenesis and are supposed to act as biomarkers for biomedical applications [18].

Lung cancer is the most common cancer in whole population from China, especially in males. Although genetic polymorphisms associated with lung cancer have been discussed previously, the effects of 3′UTR genetic variants in Chinese Han population are seldom reported. We thus selected SNPs in 3′UTR with MAF>0.05 in* IL-16*,* CYP24A1*, and* FBN1* in order to investigate their potential roles in lung oncogenesis with statistical approaches. The relationship of genetic polymorphisms and lung cancer risk was estimated by frequency distribution analysis between patients and healthy controls. Our research could further yield new insights on polymorphic regulatory sites in 3′UTR of gene in lung cancer tumorigenesis.

## 2. Materials and Methods

### 2.1. Study Subjects

A case-control study involving a Chinese Han study population of 322 lung cancer patients and 384 healthy controls was conducted at the Second Affiliated Hospital of Hainan Medical University. The lung diagnoses of all patients were confirmed by pathological analysis. Tumor stages were examined according to the tumor-node-metastasis (TNM) classification. The exclusion criteria included self-reported cancer history and previous radiotherapy or chemotherapy. There were no sex, age, or stage restrictions for cases. None of the healthy control subjects had any chronic or severe malignancy, autoimmune, or pulmonary diseases. All the participants were genetically unrelated ethnic Han Chinese. Personal details (age, gender, smoking, and drinking status) and clinical data (pathological type, TNM stage, and lymph node metastasis) were retrieved by clinicians from medical records. Individuals who smoked more than one year or drank over three times a week more than six months were defined as smokers or drinkers and otherwise were considered as nonsmokers or nondrinkers.

### 2.2. Ethics Statement

All participants were informed both in writing and verbally of the procedures and purpose of the study, and they signed informed consent documents. The protocols for this study were approved by the Ethical Committee of the Second Affiliated Hospital of Hainan Medical University, and they complied with the World Medical Association Declaration of Helsinki. All the subsequent research analyses were carried out in accordance with the approved guidelines and regulations.

### 2.3. SNP Genotyping

In this study, we selected three candidate polymorphisms including* IL-16* rs859,* CYP24A1* rs4809957, and* FBN1* rs1042078 in order to detect their effects on lung cancer risk for Chinese Han individuals. Each SNP had minor allele frequency (MAF) of > 5% in the HapMap of the Chinese Han CHB population. Genomic DNA was isolated from whole blood samples using the GoldMag-Mini Whole Blood Genomic DNA Purification Kit (GoldMag Co. Ltd., Xi'an City, China) according to the manufacturer's instructions. Quantification of the extracted DNA was performed using NanoDrop2000 (Thermo Scientific, Waltham, Massachusetts, USA) at a wavelength of 260 nm. The multiplexed SNP Mass EXTENDED assay was designed using Sequenom MassArray Assay Design 3.0 Software [19]. SNP genotyping was performed using the Sequenom Mass Array RS1000 (Sequenom, San Diego, CA) [19]. Data analysis was performed using Sequenom Typer 4.0 software (Sequenom) [19, 20]. The PCR primers for the three SNPs are shown in Supplementary Table S1.

### 2.4. Statistical Analyses

We used Microsoft Excel and SPSS 19.0 (SPSS, Chicago, IL, USA) for statistical analyses. In this study, the control genotype frequency for each SNP was analyzed by exact test to evaluate their departure from Hardy-Weinberg equilibrium (HWE). Pearson Chi-Square test was applied to detect the allele and genotype frequency distribution differences between patients and controls. All *P* values were two-sided, and* P* ≤ 0.05 was considered statistically significant. Moreover, four genetic models (codominant, dominant, recessive and log-additive) were generated using PLINK software (http://pngu.mgh.harvard.edu/purcell/plink/) to estimate the relationship between each SNP and lung cancer risk. Unconditional logistic regression analysis was used to obtain the odds ratio (OR) and 95% confidence interval (CI) for all variants [21, 22].

## 3. Results

A convenience sample of 322 lung cancer patients (245 males and 77 females) was enrolled in this study as well as 384 healthy controls (278 males and 106 females), with a mean age of 59.00 ± 9.83 and 51.16 ± 11.49 respectively (Table 1). A significant difference in age distribution was detected between cases and controls (*P* < 0.05), but adjusted analysis was performed in the following statistics. Moreover, stratified analysis for age was also performed to eliminate the influence. Additionally, distribution of the selected characteristics among the controls and cases are summarized in Table 1.

*IL-16* rs859,* CYP24A1* rs4809957, and* FBN1* rs1042078 were selected SNPs located in 3′UTR region and specific information of these variants is provided in Table 2. All of the three SNPs in controls were in compliance with the Hardy-Weinberg equilibrium (HWE) (*P* > 0.05). As depicted in Table 2, the minor allele frequencies of the selected variants ranged from approximately 37.9% to 53.3% and 38.7% to 48.7% for cases and controls, respectively. There was no difference in allele frequency between these two study groups (*P* > 0.05).

Genetic model analyses of the three SNPs were conducted in this study and the results are listed in Table 3. Compared with the genotype “A/A-G/A”,* IL-16* rs859 “G/G” showed a decrease in lung cancer susceptibility with OR of 0.65 (95% CI = 0.44-0.96,* P* = 0.029) in recessive model after adjusting for age, gender, smoking status, and alcohol drinking status. Moreover, reduced risk of lung cancer was also detected in log-additive model (adjusted OR = 0.76, 95% CI = 0.60-0.96,* P* =0.019). However, there were no significant differences between the two study sets at* CYP24A1* rs4809957 and* FBN1* rs1042078 with or without adjustment (*P* > 0.05).

Furthermore, we performed a stratified study in order to explore the significant correlations between the SNPs and lung cancer development in subgroups with different age or gender. Stratified analysis by age revealed that the* IL-16* SNPs rs859 was statistically significant in correlation to lung cancer risk in the cohort aged older than 50. In Table 4, “G/G” genotype reduced the lung cancer risk by about 52% when compared with “A/A” carriers (G/G: adjusted OR = 0.48, 95% CI = 0.28-0.83,* P* = 0.021). Recessive model (ref: A/A-G/A, adjusted OR = 0.56, 95% CI = 0.36-0.87,* P* = 0.0089) and log-additive model (adjusted OR = 0.69, 95% CI = 0.52-0.91,* P* =0.0071) analyses exhibited decreased risk association of rs859 with lung cancer (Table 4), whereas there was of no significance among individuals aged younger than 50 (Supplementary Table S2;* P* > 0.05). Likewise,* IL-16* rs859 exerted a protective role in lung cancer development among males (recessive model: ref: A/A-G/A, adjusted OR = 0.62, 95% CI = 0.39-0.99,* P* = 0.045; log-additive model: adjusted OR = 0.73, 95% CI = 0.55-0.97,* P* =0.029) (Table 5). However, there was no relationship existed between the selected SNPs and lung cancer risk in females (Supplementary Table S3). Unfortunately, stratified results for gender and age on* CYP24A1* rs4809957 and* FBN1* rs1042078 did not uncover any associations of these SNPs with lung cancer susceptibility in Chinese Han population (*P* > 0.05).

Stratified analyses for pathological type suggested a significant association of* IL-16* rs859 with both lung adenocarcinoma (Table 6) and lung small cell carcinoma (Table 7). As presented in Table 6, the “G/G” genotype was found to be significantly associated with decreased risk of lung adenocarcinoma risk in codominant model (ref: A/A, adjusted OR = 0.49, 95% CI = 0.27-0.90,* P* = 0.035) and recessive model (ref: A/A-G/A, adjusted OR = 0.53, 95% CI = 0.32-0.87,* P* = 0.010). The protective effect of rs859 was also detected in log-additive model (adjusted OR = 0.71, 95% CI = 0.53-0.95,* P* =0.022). In addition, our findings (Table 7) indicated that the polymorphisms of rs859 also altered the predisposition of individuals to lung small cell carcinoma (dominant model: ref: A/A, adjusted OR = 0.53, 95% CI = 0.29-0.98,* P* = 0.047; log-additive model: adjusted OR = 0.67, 95% CI = 0.46-0.99,* P* =0.044). There was no obvious evidence for rs859 related to lung squamous cell carcinoma risk (Supplementary Table S4), TNM staging (Supplementary Table S5), and lymph node metastasis (Supplementary Table S6); however,* CYP24A1 *rs4809957 appeared to be correlated with TNM staging and lymph node metastasis of lung cancer in different genetic models (*P* > 0.05; Supplementary Table S5-S6).

We further investigated the potential interactions between the three selected SNPs and smoking or drinking behavior in lung carcinogenesis. When stratified by drinking status,* IL-16* rs859 was found to modulate the susceptibility to lung cancer among individuals without drinking history (Table 8; log-additive model: adjusted OR = 0.74, 95% CI = 0.55-0.98,* P* = 0.036). After adjustment for confounding factors, there were still no statistically significant findings for all variants among drinkers, smokers, and nonsmokers (*P* > 0.05; Supplementary Table S7-S9).

## 4. Discussion

IL-16 is a multifunctional proinflammatory cytokine and acts as a primary chemotactin for CD4^+^ T-lymphocytes, monocytes, eosinophils, and dendritic cells [23]. The secretions of these cells could affect inflammation-related lung diseases, such as asthma, allergic rhinitis and idiopathic pulmonary fibrosis [23, 24]. Furthermore, several studies have indicated that variations in* IL-16* are associated with cancer risk. A current meta-analysis has concluded a positive relationship between* IL-16* rs1131445 C/T and cancer risk in Asian populations [25]. And the contribution of* IL-16* rs4778889 has been demonstrated in correlation to renal cell cancer in a Chinese population [26]. In this study, the polymorphism rs859 in 3′UTR of* IL-16* has been demonstrated in correlation to lung cancer for the first time. As a main result of our research,* IL-16* 3′UTR variant rs859, was associated with lung cancer risk in Chinese Han population. It was noteworthy that this relationship still survived among the people older than 50, males, and nondrinkers after stratification, which suggest a promising marker for lung cancer risk assessment and prevention in these cohorts. Our findings also uncovered significant associations of rs859 with susceptibility to lung adenocarcinoma and lung small cell carcinoma. As the fact that lung cancer is the most common cancer in Chinese population, especially in males, individual genotype detection of rs859 is worthy of recommendation for high risk group in order to provide them with effective clinical supervision and minimize the cancer susceptibility.

Translation processes of mRNAs could be inhibited by miRNAs with sequence complementarity and thermodynamics binding within 3′UTR. In this case, there is a reduction of protein and dysregulation of downstream activities which thereby lead to tumorigenesis [15]. 3′UTR of mRNAs have been underscored as potential sources of functional polymorphisms with possibly influences on cancer development. Mounting evidence has uncovered that genetic alterations at miRNA target sequences contribute to carcinogenesis. Effects of these variations have been discussed in esophageal, gastric, colorectal, breast, papillary thyroid, and nasopharyngeal cancer, with applicable clinical values as genetic markers of cancer risk, as well as biomarkers of cancer subtype, outcome, and response to therapy [14, 16, 27]. IL-16 resulting from 3′UTR variant rs859 might cause the imperfect recognition and binding of miRNAs significantly and hence alter the level of its products in inflammatory responses. Since IL-16 has been proved associated with cancer progression and susceptibility [28, 29], the abnormal expression of IL-16 because of 3′UTR alterations might result in the disordered modulation implicated in the generation and development of lung cancer. However, a further interaction assay is necessary to better characterize the role of rs859 in miRNA regulation and elucidate the underlying molecular mechanisms of functional SNPs in tumorigenesis of lung cancer. Additionally, various well-discussed functions of untranslated region also suggest the other putative effects of* IL-16* rs859 in miRNA stability, protein translation, and localization, which might be implicated in lung cancer development as well.

According to the previous research, genetic association studies have been carried out between gene polymorphisms and lung cancer risk in Chinese Han population [30–35]. Due to the fact that most GWAS reported risk associated variants are found in noncoding regions and polymorphisms in untranslated region are correlated to differential gene expression patterns, our work focused on the association of SNPs resided in gene 3′UTR with lung cancer risk for the first time. And significant associated evidences have been detected between* IL-16* rs859 and lung cancer susceptibility. However, limitations should be acknowledged in this study. First, the age difference still exists between the lung cancer cases and healthy controls, because it is difficult to find elder subjects with eligible health conditions. Although the age-adjustment and age-stratified analysis have enhanced the accuracy and significance of rs859 in* IL-16*, the potential roles of other two SNPs might be concealed in the age-stratified subgroups, especially among individuals aged younger than 50 years owing to the relatively small sample size of the cases (156 controls and 58 cases). Second,* CYP24A1* rs4809957 was demonstrated to be only related to the TNM staging and lymph node metastasis of lung cancer in this work. The possible risk association of this variant with lung cancer needs to be further studied. Third, the mechanism by which the alleles “A” and “G” at rs859 influence the individual lung risk should be testified by experiment. Thus, further prospective studies with well-matched population and biological functional experiments could reinforce the statistical power and achieve a profound understanding of our results.

## 5. Conclusions

In this study, the polymorphisms in 3′UTR of* IL-16* have been demonstrated in correlation to lung cancer for the first time. Our results suggested a significant relationship between* IL-16* 3′UTR rs859 and lung cancer risk in a Chinese Han population. Our results yield a new insight on* IL-16* SNPs in mRNA untranslated region and provide possible candidate for lung cancer risk assessment in Chinese Han population.

## Figures and Tables

**Table 1:** Distribution of the selected characteristics in lung cancer cases and controls.

Variables	Cases (N = 322)	Controls (N = 384)	*P* value
Gender			> 0.05^a^
Male	245 (76.1%)	278 (72.4%)	
Female	77 (23.9%)	106 (27.6%)	
Age (years)	59.00	51.16	< 0.05^b^
Standard Deviation	9.83	11.49	
Smoking Status			< 0.001^a^
Smokers	182 (56.5%)	164 (42.7%)	
Non-smokers	140 (43.5%)	220 (57.3%)	
Alcohol Drinking Status			< 0.001^a^
Ever	93 (28.9%)	169 (44.0%)	
Never	229 (71.1%)	215 (56.0%)	
Pathological Type			
Adenocarcinoma	150 (46.6%)		
Squamous Cell Carcinoma	98 (30.4%)		
Small Cell Carcinoma	74 (23.0%)		
TNM Stage			
I-II Stage	75 (23.3%)		
III-IV Stage	213 (66.1%)		
Unknown	34 (10.6%)		
Lymph Node Metastasis			
Positive Cases	195 (60.6%)		
Negative Cases	127 (39.4%)		

*P* ^a^-value: *P*-value obtained from Chi-squared test.

*P* ^b^-value: *P*-value obtained from independent sample *t*-test.

**Table 2:** Basic information and allele frequencies of the selected polymorphisms in 3′UTR of *IL-16*, C*YP24A1*, and *FBN1*.

SNP	Chromosome	Position	Alleles	Gene	Position	Minor Allele Frequency		HWE	OR	*P* ^b^-value
			A<B			Case	Control	*P* ^a^-value	(95% CI)	
rs859	chr15	81601322	A<G	IL-16	UTR-3	0.533	0.487	0.839	1.20 (0.97-1.48)	0.087
rs4809957	chr20	52771171	A<G	CYP24A1	UTR-3	0.379	0.387	0.830	0.97 (0.78-1.20)	0.763
rs1042078	chr15	48702873	G<A	FBN1	UTR-3	0.452	0.454	0.607	0.99 (0.80-1.22)	0.923

SNP: Single Nucleotide Polymorphism; OR: Odd Ratio; 95% CI: 95% Confidence Interval; HWE: Hardy-Weinberg Equilibrium.

HWE *P*^a^-value: *P*-values obtained from exact test.

*P* ^b^-value: *P*-values obtained from Chi-squared test.

**Table 3:** Genetic model analyses of *IL-16*-rs859, *CYP24A1*-rs4809957, and *FBN1*-rs1042078with lung cancer risk.

Gene	SNP	Model	Genotype	Control	Case	Crude Analysis		Adjusted Analysis	
				(N = 384)	(N = 322)	OR (95% CI)	*P* ^a^-value	OR (95% CI)	*P* ^b^-value
IL-16 (N = 706)	rs859 (call rate 99.86%)	Codominant	A/A	92 (24.0%)	89 (27.7%)	1.00	0.200	1.00	0.058
			G/A	190 (49.5%)	164 (51.1%)	0.89 (0.62-1.28)		0.82 (0.55-1.22)	
			G/G	102 (26.6%)	68 (21.2%)	0.69 (0.45-1.05)		0.57 (0.36-0.91)	
		Dominant	A/A	92 (24.0%)	89 (27.7%)	1.00	0.250	1.00	0.100
			G/A-G/G	292 (76.0%)	232 (72.3%)	0.82 (0.59-1.15)		0.73 (0.50-1.06)	
		Recessive	A/A-G/A	282 (73.4%)	253 (78.8%)	1.00	0.095	1.00	***0.029***
			G/G	102 (26.6%)	68 (21.2%)	0.74 (0.52-1.06)		**0.65 (0.44-0.96)**	
		Log-additive	---	---	---	0.83 (0.67-1.03)	0.086	**0.76 (0.60-0.96)**	***0.019***

CYP24A1 (N = 706)	rs4809957 (call rate 100%)	Codominant	G/G	143 (37.2%)	124 (38.5%)	1.00	0.940	1.00	0.560
			G/A	185 (48.2%)	152 (47.2%)	0.95 (0.69-1.31)		0.89 (0.62-1.27)	
			A/A	56 (14.6%)	46 (14.3%)	0.95 (0.60-1.50)		0.76 (0.46-1.27)	
		Dominant	G/G	143 (37.2%)	124 (38.5%)	1.00	0.730	1.00	0.370
			G/A-A/A	241 (62.8%)	198 (61.5%)	0.95 (0.70-1.29)		0.86 (0.61-1.20)	
		Recessive	G/G-G/A	328 (85.4%)	276 (85.7%)	1.00	0.910	1.00	0.400
			A/A	56 (14.6%)	46 (14.3%)	0.98 (0.64-1.49)		0.82 (0.51-1.31)	
		Log-additive	---	---	---	0.97 (0.78-1.20)	0.760	0.88 (0.69-1.12)	0.280

FBN1 (N = 706)	rs1042078 (call rate 100%)	Codominant	A/A	117 (30.5%)	85 (26.4%)	1.00	0.065	1.00	0.200
			A/G	185 (48.2%)	183 (56.8%)	1.36 (0.96-1.92)		1.27 (0.86-1.86)	
			G/G	82 (21.4%)	54 (16.8%)	0.91 (0.58-1.41)		0.88 (0.54-1.44)	
		Dominant	A/A	117 (30.5%)	85 (26.4%)	1.00	0.230	1.00	0.450
			A/G-G/G	267 (69.5%)	237 (73.6%)	1.22 (0.88-1.70)		1.15 (0.80-1.66)	
		Recessive	A/A-A/G	302 (78.7%)	268 (83.2%)	1.00	0.120	1.00	0.190
			G/G	82 (21.4%)	54 (16.8%)	0.74 (0.51-1.09)		0.76 (0.50-1.15)	
		Log-additive	---	---	---	0.99 (0.80-1.23)	0.920	0.97 (0.76-1.23)	0.790

SNP: Single Nucleotide Polymorphism; OR: Odds Ratio; 95% CI: 95% Confidence Interval.

*P* ^a^-value: *P*-values calculated by unconditional logistic regression analysis.

*P* ^b^-value: *P*-values calculated by unconditional logistic regression analysis with adjustment for age, gender, smoking status, and alcohol drinking status.

Bold italics indicates the statistical significance (*P *< 0.05).

**Table 4:** The relationship between *IL-16-*rs859*, CYP24A1-*rs4809957,and *FBN1-*rs1042078 and lung cancer risk among the people aged older than 50.

Gene	SNP	Model	Genotype	Control	Case	Crude Analysis		Adjusted Analysis	
				(N = 228)	(N = 264)	OR (95% CI)	*P* ^a^-value	OR (95% CI)	*P* ^b^-value
IL-16 (N = 492)	rs859 (call rate 100%)	Codominant	A/A	44 (19.3%)	72 (27.3%)	1.00	***0.009***	1.00	***0.021***
			G/A	109 (47.8%)	135 (51.1%)	0.76 (0.48-1.19)		0.79 (0.49-1.29)	
			G/G	75 (32.9%)	57 (21.6%)	**0.46 (0.28-0.77)**		**0.48 (0.28-0.83)**	
		Dominant	A/A	44 (19.3%)	72 (27.3%)	1.00	***0.037***	1.00	0.079
			G/A-G/G	184 (80.7%)	192 (72.7%)	**0.64 (0.42-0.98)**		0.66 (0.42-1.05)	
		Recessive	A/A-G/A	153 (67.1%)	207 (78.4%)	1.00	***0.0048***	1.00	***0.0089***
			G/G	75 (32.9%)	57 (21.6%)	**0.56 (0.38-0.84)**		**0.56 (0.36-0.87)**	
		Log-additive	---	---	---	**0.68 (0.53-0.87)**	***0.0026***	**0.69 (0.52-0.91)**	***0.0071***

CYP24A1 (N = 492)	rs4809957 (call rate 100%)	Codominant	G/G	84 (36.8%)	97 (36.7%)	1.00	1.000	1.00	0.780
			G/A	111 (48.7%)	128 (48.5%)	1.00 (0.68-1.47)		0.98 (0.64-1.48)	
			A/A	33 (14.5%)	39 (14.8%)	1.02 (0.59-1.77)		0.81 (0.44-1.48)	
		Dominant	G/G	84 (36.8%)	97 (36.7%)	1.00	0.980	1.00	0.750
			G/A-A/A	144 (63.2%)	167 (63.3%)	1.00 (0.70-1.45)		0.94 (0.63-1.40)	
		Recessive	G/G-G/A	195 (85.5%)	225 (85.2%)	1.00	0.930	1.00	0.490
			A/A	33 (14.5%)	39 (14.8%)	1.02 (0.62-1.69)		0.82 (0.47-1.43)	
		Log-additive	---	---	---	1.01 (0.78-1.31)	0.950	0.92 (0.69-1.22)	0.560

FBN1 (N = 492)	rs1042078 (call rate 100%)	Codominant	A/A	69 (30.3%)	72 (27.3%)	1.00	0.250	1.00	0.380
			A/G	114 (50.0%)	151 (57.2%)	1.27 (0.84-1.91)		1.18 (0.75-1.84)	
			G/G	45 (19.7%)	41 (15.5%)	0.87 (0.51-1.49)		0.82 (0.46-1.46)	
		Dominant	A/A	69 (30.3%)	72 (27.3%)	1.00	0.460	1.00	0.740
			A/G-G/G	159 (69.7%)	192 (72.7%)	1.16 (0.78-1.71)		1.08 (0.70-1.65)	
		Recessive	A/A-A/G	183 (80.3%)	223 (84.5%)	1.00	0.220	1.00	0.230
			G/G	45 (19.7%)	41 (15.5%)	0.75 (0.47-1.19)		0.74 (0.45-1.22)	
		Log-additive	---	---	---	0.97 (0.75-1.27)	0.840	0.94 (0.70-1.25)	0.650

SNP: Single Nucleotide Polymorphism; OR: Odds Ratio; 95% CI: 95% Confidence Interval.

*P* ^a^-value: *P*-values calculated by unconditional logistic regression analysis.

*P* ^b^-value: *P*-values calculated by unconditional logistic regression analysis with adjustment for age, gender, smoking status, and alcohol drinking status.

Bold italics indicates the statistical significance (*P *< 0.05).

**Table 5:** The relationship between *IL-16*-rs859, *CYP24A1*-rs4809957, and *FBN1*-rs1042078 and lung cancer risk in males.

Gene	SNP	Model	Genotype	Control	Case	Crude Analysis		Adjusted Analysis	
				(N = 278)	(N = 245)	OR (95% CI)	*P* ^a^-value	OR (95% CI)	*P* ^b^-value
IL-16 (N = 523)	rs859 (call rate 99.81%)	Codominant	A/A	67 (24.1%)	71 (29.1%)	1.00	0.190	1.00	0.084
			G/A	137 (49.3%)	123 (50.4%)	0.85 (0.56-1.28)		0.79 (0.49-1.28)	
			G/G	74 (26.6%)	50 (20.5%)	0.64 (0.39-1.04)		0.53 (0.30-0.94)	
		Dominant	A/A	67 (24.1%)	71 (29.1%)	1.00	0.200	1.00	0.120
			G/A-G/G	211 (75.9%)	173 (70.9%)	0.77 (0.52-1.14)		0.70 (0.44-1.10)	
		Recessive	A/A-G/A	204 (73.4%)	194 (79.5%)	1.00	0.100	1.00	***0.045***
			G/G	74 (26.6%)	50 (20.5%)	0.71 (0.47-1.07)		**0.62 (0.39-0.99)**	
		Log-additive	---	---	---	0.80 (0.63-1.02)	0.073	**0.73 (0.55-0.97)**	***0.029***

CYP24A1 (N = 523)	rs4809957 (call rate 100%)	Codominant	G/G	98 (35.2%)	90 (36.7%)	1.00	0.810	1.00	0.280
			G/A	136 (48.9%)	121 (49.4%)	0.97 (0.66-1.41)		0.91 (0.59-1.41)	
			A/A	44 (15.8%)	34 (13.9%)	0.84 (0.49-1.43)		0.61 (0.33-1.13)	
		Dominant	G/G	98 (35.2%)	90 (36.7%)	1.00	0.720	1.00	0.390
			G/A-A/A	180 (64.8%)	155 (63.3%)	0.94 (0.66-1.34)		0.83 (0.55-1.26)	
		Recessive	G/G-G/A	234 (84.2%)	211 (86.1%)	1.00	0.530	1.00	0.120
			A/A	44 (15.8%)	34 (13.9%)	0.86 (0.53-1.39)		0.64 (0.36-1.13)	
		Log-additive	---	---	---	0.93 (0.72-1.20)	0.570	0.81 (0.60-1.09)	0.160

FBN1 (N = 523)	rs1042078 (call rate 100%)	Codominant	A/A	85 (30.6%)	65 (26.5%)	1.00	0.220	1.00	0.280
			A/G	138 (49.6%)	140 (57.1%)	1.33 (0.89-1.98)		1.31 (0.83-2.08)	
			G/G	55 (19.8%)	40 (16.3%)	0.95 (0.57-1.60)		0.90 (0.50-1.62)	
		Dominant	A/A	85 (30.6%)	65 (26.5%)	1.00	0.310	1.00	0.440
			A/G-G/G	193 (69.4%)	180 (73.5%)	1.22 (0.83-1.79)		1.19 (0.77-1.85)	
		Recessive	A/A-A/G	223 (80.2%)	205 (83.7%)	1.00	0.300	1.00	0.270
			G/G	55 (19.8%)	40 (16.3%)	0.79 (0.50-1.24)		0.75 (0.45-1.25)	
		Log-additive	---	---	---	1.01 (0.79-1.31)	0.920	0.98 (0.73-1.31)	0.900

SNP: Single Nucleotide Polymorphism; OR: Odds Ratio; 95% CI: 95% Confidence Interval.

*P* ^a^-value: *P*-values calculated by unconditional logistic regression analysis.

*P* ^b^-value: *P*-values calculated by unconditional logistic regression analysis with adjustment for age, smoking status, and alcohol drinking status.

Bold italics indicates the statistical significance (*P* < 0.05).

**Table 6:** The relationship between *IL-16-*rs859*, CYP24A1-*rs4809957,and *FBN1-*rs1042078 and lung adenocarcinoma risk.

Gene	SNP	Model	Genotype	Control	Case	Crude Analysis		Adjusted Analysis	
				(N = 384)	(N = 150)	OR (95% CI)	*P* ^a^-value	OR (95% CI)	*P* ^b^-value
IL-16 (N = 534)	rs859 (call rate 100%)	Codominant	A/A	92 (24.0%)	39 (26.0%)	1.00	0.150	1.00	***0.035***
			G/A	190 (49.5%)	83 (55.3%)	1.03 (0.65-1.62)		0.90 (0.55-1.48)	
			G/G	102 (26.6%)	28 (18.7%)	0.65 (0.37-1.14)		**0.49 (0.27-0.90)**	
		Dominant	A/A	92 (24.0%)	39 (26.0%)	1.00	0.620	1.00	0.240
			G/A-G/G	292 (76.0%)	111 (74.0%)	0.90 (0.58-1.38)		0.75 (0.47-1.21)	
		Recessive	A/A-G/A	282 (73.4%)	122 (81.3%)	1.00	0.052	1.00	***0.010***
			G/G	102 (26.6%)	28 (18.7%)	0.63 (0.40-1.01)		**0.53 (0.32-0.87)**	
		Log-additive	---	---	---	0.82 (0.62-1.07)	0.140	**0.71 (0.53-0.95)**	***0.022***

CYP24A1 (N = 534)	rs4809957 (call rate 100%)	Codominant	G/G	143 (37.2%)	64 (42.7%)	1.00	0.270	1.00	0.210
			G/A	185 (48.2%)	71 (47.3%)	0.86 (0.57-1.28)		0.81 (0.53-1.25)	
			A/A	56 (14.6%)	15 (10.0%)	0.60 (0.32-1.14)		0.55 (0.28-1.09)	
		Dominant	G/G	143 (37.2%)	64 (42.7%)	1.00	0.250	1.00	0.180
			G/A-A/A	241 (62.8%)	86 (57.3%)	0.80 (0.54-1.17)		0.75 (0.50-1.14)	
		Recessive	G/G-G/A	328 (85.4%)	135 (90.0%)	1.00	0.150	1.00	0.130
			A/A	56 (14.6%)	15 (10.0%)	0.65 (0.36-1.19)		0.62 (0.32-1.17)	
		Log-additive	---	---	---	0.80 (0.60-1.06)	0.120	0.77 (0.56-1.04)	0.083

FBN1 (N = 534)	rs1042078 (call rate 100%)	Codominant	A/A	117 (30.5%)	39 (26.0%)	1.00	0.110	1.00	0.150
			A/G	185 (48.2%)	87 (58.0%)	1.41 (0.91-2.20)		1.28 (0.80-2.05)	
			G/G	82 (21.4%)	24 (16.0%)	0.88 (0.49-1.57)		0.76 (0.41-1.41)	
		Dominant	A/A	117 (30.5%)	39 (26.0%)	1.00	0.300	1.00	0.630
			A/G-G/G	267 (69.5%)	111 (74.0%)	1.25 (0.82-1.91)		1.12 (0.71-1.76)	
		Recessive	A/A-A/G	302 (78.7%)	126 (84.0%)	1.00	0.160	1.00	0.099
			G/G	82 (21.4%)	24 (16.0%)	0.70 (0.43-1.16)		0.65 (0.38-1.10)	
		Log-additive	---	---	---	0.98 (0.75-1.29)	0.890	0.91 (0.68-1.22)	0.530

SNP: Single Nucleotide Polymorphism; OR: Odds Ratio; 95% CI: 95% Confidence Interval.

*P* ^a^-value: *P*-values calculated by unconditional logistic regression analysis.

*P* ^b^-value: *P*-values calculated by unconditional logistic regression analysis with adjustment for age, gender, smoking status, and alcohol drinking status.

Bold italics indicates the statistical significance (*P* < 0.05).

**Table 7:** The relationship between *IL-16-*rs859*, CYP24A1-*rs4809957,and *FBN1-*rs1042078 and lung small cell carcinoma risk.

Gene	SNP	Model	Genotype	Control	Case	Crude Analysis		Adjusted Analysis	
				(N = 384)	(N = 74)	OR (95% CI)	*P* ^a^-value	OR (95% CI)	*P* ^b^-value
IL-16 (N = 458)	rs859 (call rate 99.78%)	Codominant	A/A	92 (24.0%)	22 (30.1%)	1.00	0.480	1.00	0.110
			G/A	190 (49.5%)	35 (48.0%)	0.77 (0.43-1.39)		0.58 (0.30-1.11)	
			G/G	102 (26.6%)	16 (21.9%)	0.66 (0.32-1.32)		0.46 (0.21-0.99)	
		Dominant	A/A	92 (24.0%)	22 (30.1%)	1.00	0.270	1.00	***0.047***
			G/A-G/G	292 (76.0%)	51 (69.9%)	0.73 (0.42-1.27)		**0.53 (0.29-0.98)**	
		Recessive	A/A-G/A	282 (73.4%)	57 (78.1%)	1.00	0.400	1.00	0.200
			G/G	102 (26.6%)	16 (21.9%)	0.78 (0.43-1.41)		0.66 (0.35-1.26)	
		Log-additive	---	---	---	0.81 (0.57-1.15)	0.230	**0.67 (0.46-0.99)**	***0.044***

CYP24A1 (N = 458)	rs4809957 (call rate 100%)	Codominant	G/G	143 (37.2%)	28 (37.8%)	1.00	0.770	1.00	0.710
			G/A	185 (48.2%)	33 (44.6%)	0.91 (0.53-1.58)		0.88 (0.48-1.59)	
			A/A	56 (14.6%)	13 (17.6%)	1.19 (0.57-2.45)		1.21 (0.55-2.64)	
		Dominant	G/G	143 (37.2%)	28 (37.8%)	1.00	0.920	1.00	0.860
			G/A-A/A	241 (62.8%)	46 (62.2%)	0.97 (0.58-1.63)		0.95 (0.55-1.66)	
		Recessive	G/G-G/A	328 (85.4%)	61 (82.4%)	1.00	0.520	1.00	0.480
			A/A	56 (14.6%)	13 (17.6%)	1.25 (0.64-2.42)		1.30 (0.64-2.65)	
		Log-additive	---	---	---	1.02 (0.69-1.64)	0.690	1.05 (0.71-1.55)	0.800

FBN1 (N = 458)	rs1042078 (call rate 100%)	Codominant	A/A	117 (30.5%)	20 (27.0%)	1.00	0.150	1.00	0.160
			A/G	185 (48.2%)	44 (59.5%)	1.39 (0.78-2.48)		1.30 (0.70-2.41)	
			G/G	82 (21.4%)	10 (13.5%)	0.71 (0.32-1.60)		0.63 (0.26-1.49)	
		Dominant	A/A	117 (30.5%)	20 (27.0%)	1.00	0.550	1.00	0.780
			A/G-G/G	267 (69.5%)	54 (73.0%)	1.18 (0.68-2.07)		1.09 (0.60-1.98)	
		Recessive	A/A-A/G	302 (78.7%)	64 (86.5%)	1.00	0.110	1.00	0.084
			G/G	82 (21.4%)	10 (13.5%)	0.58 (0.28-1.17)		0.53 (0.25-1.13)	
		Log-additive	---	---	---	0.91 (0.64-1.31)	0.620	0.85 (0.58-1.26)	0.430

SNP: Single Nucleotide Polymorphism; OR: Odds Ratio; 95% CI: 95% Confidence Interval.

*P* ^a^-value: *P*-values calculated by unconditional logistic regression analysis.

*P* ^b^-value: *P*-values calculated by unconditional logistic regression analysis with adjustment for age, gender, smoking status, and alcohol drinking status.

Bold italics indicates the statistical significance (*P* < 0.05).

**Table 8:** The relationship between *IL-16-*rs859*, CYP24A1-*rs4809957,and *FBN1-*rs1042078 and lung cancer risk among nondrinkers.

Gene	SNP	Model	Genotype	Control	Case	Crude Analysis		Adjusted Analysis	
				(N = 215)	(N = 229)	OR (95% CI)	*P* ^a^-value	OR (95% CI)	*P* ^b^-value
IL-16 (N = 444)	rs859 (call rate 99.77%)	Codominant	A/A	47 (21.9%)	62 (27.2%)	1.00	0.200	1.00	0.110
			G/A	109 (50.7%)	118 (51.8%)	0.82 (0.52-1.30)		0.78 (0.48-1.27)	
			G/G	59 (27.4%)	48 (21.1%)	0.62 (0.36-1.06)		0.54 (0.31-0.96)	
		Dominant	A/A	47 (21.9%)	62 (27.2%)	1.00	0.190	1.00	0.130
			G/A-G/G	168 (78.1%)	166 (72.8%)	0.75 (0.48-1.16)		0.70 (0.44-1.11)	
		Recessive	A/A-G/A	156 (72.6%)	180 (79.0%)	1.00	0.120	1.00	0.062
			G/G	59 (27.4%)	48 (21.1%)	0.71 (0.46-1.09)		0.64 (0.40-1.03)	
		Log-additive	---	---	---	0.80 (0.63-1.09)	0.056	**0.74 (0.55-0.98)**	***0.036***

CYP24A1 (N = 444)	rs4809957 (call rate 100%)	Codominant	G/G	78 (36.3%)	89 (38.9%)	1.00	0.650	1.00	0.550
			G/A	106 (49.3%)	103 (45.0%)	0.85 (0.57-1.28)		0.79 (0.51-1.21)	
			A/A	31 (14.4%)	37 (16.2%)	1.05 (0.59-1.84)		0.88 (0.48-1.61)	
		Dominant	G/G	78 (36.3%)	89 (38.9%)	1.00	0.570	1.00	0.300
			G/A-A/A	137 (63.7%)	140 (61.1%)	0.90 (0.61-1.32)		0.81 (0.54-1.21)	
		Recessive	G/G-G/A	184 (85.6%)	192 (83.8%)	1.00	0.610	1.00	0.980
			A/A	31 (14.4%)	37 (16.2%)	1.14 (0.68-1.92)		1.01 (0.58-1.75)	
		Log-additive	---	---	---	0.98 (0.75-1.29)	0.900	0.90 (0.68-1.20)	0.480

FBN1 (N = 444)	rs1042078 (call rate 100%)	Codominant	A/A	63 (29.3%)	61 (26.6%)	1.00	0.058	1.00	0.210
			A/G	100 (46.5%)	130 (56.8%)	1.34 (0.87-2.08)		1.18 (0.74-1.87)	
			G/G	52 (24.2%)	38 (16.6%)	0.75 (0.44-1.30)		0.74 (0.42-1.32)	
		Dominant	A/A	63 (29.3%)	61 (26.6%)	1.00	0.530	1.00	0.880
			A/G-G/G	152 (70.7%)	168 (73.4%)	1.14 (0.75-1.73)		1.04 (0.67-1.61)	
		Recessive	A/A-A/G	163 (75.8%)	191 (83.4%)	1.00	0.046	1.00	0.110
			G/G	52 (24.2%)	38 (16.6%)	0.62 (0.39-1.00)		0.67 (0.41-1.09)	
		Log-additive	---	---	---	0.90 (0.69-1.18)	0.450	0.88 (0.66-1.18)	0.400

SNP: Single Nucleotide Polymorphism; OR: Odds Ratio; 95% CI: 95% Confidence Interval.

*P* ^a^-value: *P*-values calculated by unconditional logistic regression analysis.

*P* ^b^-value: *P*-values calculated by unconditional logistic regression analysis with adjustment for age, gender, and smoking status.

Bold italics indicates the statistical significance (*P* < 0.05).

## Data Availability

The data used to support the findings of this study are available from the corresponding author upon request.

## Supplementary Materials

Supplementary Materials PCR primers used in this study are listed in Supplementary Table S1. The relationships between* IL-16 *rs859,* CYP24A1* rs4809957, and* FBN1* rs1042078 and lung cancer risk among the people younger than 50 and Chinese Han females are showed in Supplementary Table S2 and Supplementary Table S3, respectively. The relationships between* IL-16* rs859,* CYP24A1* rs4809957, and* FBN1* rs1042078 and lung squamous cell carcinoma are exhibited in Supplementary Table S4. Supplementary Table S5 and Supplementary Table S6 presented the stratified analysis of* IL-16* rs859,* CYP24A1* rs4809957, and* FBN1* rs1042078 for TNM staging and lymph node metastasis. The relationships between* IL-16* rs859,* CYP24A1* rs4809957, and* FBN1* rs1042078 and lung cancer risk among drinkers are showed in Supplementary Table S7. Supplementary Table S8 and Supplementary Table S9 demonstrated the relationships between* IL-16 *rs859,* CYP24A1* rs4809957, and* FBN1* rs1042078 and lung cancer risk among smokers and nonsmokers, respectively.
