# Supplementary material for: The Impact of IL-16 3′UTR Polymorphism rs859 on Lung Carcinoma Susceptibility among Chinese Han Individuals
Source: Biomed Res Int. 2018 Dec 24;2018:8305745. doi: 10.1155/2018/8305745 (PMC6323476; doi:10.1155/2018/8305745)
Supplement: Supplementary Materials — PCR primers used in this study are listed in Supplementary Table S1. The relationships between IL-16 rs859, CYP24A1 rs4809957, and FBN1 rs1042078 and lung cancer risk among the people younger than 50 and Chinese Han females are showed in Supplementary Table S2 and Supplementary Table S3, respectively. The relationships between IL-16 rs859, CYP24A1 rs4809957, and FBN1 rs1042078 and lung squamous cell carcinoma are exhibited in Supplementary Table S4. Supplementary Table S5 and Supplementary Table S6 presented the stratified analysis of IL-16 rs859, CYP24A1 rs4809957, and FBN1 rs1042078 for TNM staging and lymph node metastasis. The relationships between IL-16 rs859, CYP24A1 rs4809957, and FBN1 rs1042078 and lung cancer risk among drinkers are showed in Supplementary Table S7. Supplementary Table S8 and Supplementary Table S9 demonstrated the relationships between IL-16 rs859, CYP24A1 rs4809957, and FBN1 rs1042078 and lung cancer risk among smokers and nonsmokers, respectively. [file 8305745.f1.docx]

Supplementary Table S1: PCR primers used in this study.

| SNP | First PCRP (5'-3') | Second PCRP (5'-3') | UEP SEQ (5'-3') |
| --- | --- | --- | --- |
| rs859 | ACGTTGGATGCCACCCAGCAAAAGGTTGTT | ACGTTGGATGCAATTTGTATCAGCTGCCCC | TGTGACTCTGCCCTTA |
| rs1042078 | ACGTTGGATGGCCATTTATGCTGACATGCC | ACGTTGGATGGTAGTCCATCAATTGAAAGC | tcagCAATTGAAAGCACATTCCC |
| rs4809957 | ACGTTGGATGAGATGCAAGACAGAACAGGC | ACGTTGGATGGCTGGTTTACACTTCACCTG | ggggaATCTTCAGGTGCTTAGAA |

**PCRP：PCR Primer; UEP：Unextended Mini Sequencing Primer.**

Supplementary Table S2: The relationship between *IL-16-*rs859*, CYP24A1-*rs4809957 and *FBN1-*rs1042078 and lung cancer risk among the people aged younger than 50.

| Gene | SNP | Model | Genotype | Control  (N = 156) | Case  (N = 58) | Crude Analysis | | Adjusted Analysis | |
| --- | --- | --- | --- | --- | --- | --- | --- | --- | --- |
|  |  |  |  |  |  | OR (95% CI) | *P*^a^-value | OR (95% CI) | *P*^b^-value |
| IL-16  (N = 214) | rs859 (call rate 99.53%) | Codominant | A/A | 48 (30.8%) | 17 (29.8%) | 1.00 | 0.950 | 1.00 | 0.820 |
|  |  |  | G/A | 81 (51.9%) | 29 (50.9%) | 1.01 (0.50-2.03) |  | 1.02 (0.46-2.28) |  |
|  |  |  | G/G | 27 (17.3%) | 11 (19.3%) | 1.15 (0.47-2.81) |  | 1.36 (0.48-3.85) |  |
|  |  | Dominant | A/A | 48 (30.8%) | 17 (29.8%) | 1.00 | 0.890 | 1.00 | 0.800 |
|  |  |  | G/A-G/G | 108 (69.2%) | 40 (70.2%) | 1.05 (0.54-2.03) |  | 1.10 (0.51-2.36) |  |
|  |  | Recessive | A/A-G/A | 129 (82.7%) | 46 (80.7%) | 1.00 | 0.740 | 1.00 | 0.530 |
|  |  |  | G/G | 27 (17.3%) | 11 (19.3%) | 1.14 (0.52-2.49) |  | 1.34 (0.54-3.32) |  |
|  |  | Log-additive | --- | --- | --- | 1.06 (0.68-1.66) | 0.780 | 1.15 (0.69-1.92) | 0.600 |
|  | | | | | | | | | |
| CYP24A1  (N = 214) | rs4809957 (call rate 100%) | Codominant | G/G | 59 (37.8%) | 27 (46.5%) | 1.00 | 0.510 | 1.00 | 0.230 |
|  |  |  | G/A | 74 (47.4%) | 24 (41.4%) | 0.71 (0.37-1.35) |  | 0.55 (0.26-1.17) |  |
|  |  |  | A/A | 23 (14.7%) | 7 (12.1%) | 0.67 (0.25-1.74) |  | 0.50 (0.16-1.51) |  |
|  |  | Dominant | G/G | 59 (37.8%) | 27 (46.5%) | 1.00 | 0.250 | 1.00 | 0.086 |
|  |  |  | G/A-A/A | 97 (62.2%) | 31 (53.5%) | 0.70 (0.38-1.28) |  | 0.54 (0.26-1.10) |  |
|  |  | Recessive | G/G-G/A | 133 (85.3%) | 51 (87.9%) | 1.00 | 0.610 | 1.00 | 0.460 |
|  |  |  | A/A | 23 (14.7%) | 7 (12.1%) | 0.79 (0.32-1.96) |  | 0.68 (0.24-1.91) |  |
|  |  | Log-additive | --- | --- | --- | 0.78 (0.50-1.22) | 0.280 | 0.66 (0.39-1.11) | 0.110 |
|  |  |  |  |  |  |  |  |  |  |
| FBN1  (N = 214) | rs1042078 (call rate 100%) | Codominant | A/A | 48 (30.8%) | 13 (22.4%) | 1.00 | 0.380 | 1.00 | 0.160 |
|  |  |  | A/G | 71 (45.5%) | 32 (55.2%) | 1.66 (0.79-3.49) |  | 1.83 (0.77-4.33) |  |
|  |  |  | G/G | 37 (23.7%) | 13 (22.4%) | 1.30 (0.54-3.13) |  | 0.86 (0.32-2.35) |  |
|  |  | Dominant | A/A | 48 (30.8%) | 13 (22.4%) | 1.00 | 0.220 | 1.00 | 0.390 |
|  |  |  | A/G-G/G | 108 (69.2%) | 45 (77.6%) | 1.54 (0.76-3.11) |  | 1.42 (0.63-3.16) |  |
|  |  | Recessive | A/A-A/G | 119 (76.3%) | 45 (77.6%) | 1.00 | 0.840 | 1.00 | 0.190 |
|  |  |  | G/G | 37 (23.7%) | 13 (22.4%) | 0.93 (0.45-1.91) |  | 0.58 (0.25-1.34) |  |
|  |  | Log-additive | --- | --- | --- | 1.15 (0.75-1.74) | 0.520 | 0.94 (0.58-1.52) | 0.800 |

**SNP: Single Nucleotide Polymorphism; OR: Odds Ratio; 95% CI: 95% Confidence Interval.**

***P*^a^-value: *P*-values calculated by unconditional logistic regression analysis.**

***P*^b^-value: *P*-values calculated by unconditional logistic regression analysis with adjustment for age, gender, smoking status and alcohol drinking status.**

Supplementary Table S3: The relationship between *IL-16*-rs859, *CYP24A1*-rs4809957 and *FBN1*-rs1042078 and lung cancer risk in females.

| Gene | SNP | Model | Genotype | Control  (N = 106) | Case  (N = 77) | Crude Analysis | | Adjusted Analysis | |
| --- | --- | --- | --- | --- | --- | --- | --- | --- | --- |
|  |  |  |  |  |  | OR (95% CI) | *P*^a^-value | OR (95% CI) | *P*^b^-value |
| IL-16  (N = 183) | rs859 (call rate 100%) | Codominant | G/G | 28 (26.4%) | 18 (23.4%) | 1.00 | 0.880 | 1.00 | 0.690 |
|  |  |  | G/A | 53 (50.0%) | 41 (53.2%) | 1.20 (0.59-2.47) |  | 1.31 (0.63-2.75) |  |
|  |  |  | A/A | 25 (23.6%) | 18 (23.4%) | 1.12 (0.48-2.61) |  | 1.43 (0.59-3.46) |  |
|  |  | Dominant | G/G | 28 (26.4%) | 18 (23.4%) | 1.00 | 0.640 | 1.00 | 0.410 |
|  |  |  | G/A-A/A | 78 (73.6%) | 59 (76.6%) | 1.18 (0.59-2.33) |  | 1.35 (0.67-2.72) |  |
|  |  | Recessive | G/G-G/A | 81 (76.4%) | 59 (76.6%) | 1.00 | 0.970 | 1.00 | 0.650 |
|  |  |  | A/A | 25 (23.6%) | 18 (23.4%) | 0.99 (0.49-1.98) |  | 1.18 (0.57-2.44) |  |
|  |  | Log-additive | --- | --- | --- | 1.06 (0.70-1.62) | 0.790 | 1.20 (0.77-1.86) | 0.420 |
|  | | | | | | | | | |
| CYP24A1  (N = 183) | rs4809957 (call rate 100%) | Codominant | G/G | 45 (42.5%) | 34 (44.2%) | 1.00 | 0.600 | 1.00 | 0.600 |
|  |  |  | G/A | 49 (46.2%) | 31 (40.3%) | 0.84 (0.44-1.58) |  | 0.79 (0.41-1.50) |  |
|  |  |  | A/A | 12 (11.3%) | 12 (15.6%) | 1.32 (0.53-3.31) |  | 1.21 (0.47-3.10) |  |
|  |  | Dominant | G/G | 45 (42.5%) | 34 (44.2%) | 1.00 | 0.820 | 1.00 | 0.650 |
|  |  |  | G/A-A/A | 61 (57.5%) | 43 (55.8%) | 0.93 (0.52-1.69) |  | 0.87 (0.47-1.60) |  |
|  |  | Recessive | G/G-G/A | 94 (88.7%) | 65 (84.4%) | 1.00 | 0.400 | 1.00 | 0.490 |
|  |  |  | A/A | 12 (11.3%) | 12 (15.6%) | 1.45 (0.61-3.42) |  | 1.37 (0.57-3.30) |  |
|  |  | Log-additive | --- | --- | --- | 1.06 (0.69-1.62) | 0.800 | 1.01 (0.65-1.56) | 0.980 |
|  |  |  |  |  |  |  |  |  |  |
| FBN1  (N = 183) | rs1042078 (call rate 100%) | Codominant | A/A | 32 (30.2%) | 20 (26.0%) | 1.00 | 0.280 | 1.00 | 0.490 |
|  |  |  | A/G | 47 (44.3%) | 43 (55.8%) | 1.46 (0.73-2.93) |  | 1.25 (0.61-2.57) |  |
|  |  |  | G/G | 27 (25.5%) | 14 (18.2%) | 0.83 (0.35-1.95) |  | 0.78 (0.33-1.88) |  |
|  |  | Dominant | A/A | 32 (30.2%) | 20 (26.0%) | 1.00 | 0.530 | 1.00 | 0.820 |
|  |  |  | A/G-G/G | 74 (69.8%) | 57 (74.0%) | 1.23 (0.64-2.38) |  | 1.08 (0.55-2.13) |  |
|  |  | Recessive | A/A-A/G | 79 (74.5%) | 63 (81.8%) | 1.00 | 0.240 | 1.00 | 0.310 |
|  |  |  | G/G | 27 (25.5%) | 14 (18.2%) | 0.65 (0.31-1.34) |  | 0.68 (0.32-1.43) |  |
|  |  | Log-additive | --- | --- | --- | 0.94 (0.62-1.42) | 0.770 | 0.91 (0.59-1.39) | 0.650 |

**SNP: Single Nucleotide Polymorphism; OR: Odds Ratio; 95% CI: 95% Confidence Interval.**

***P*^a^-value: *P*-values calculated by unconditional logistic regression analysis.**

***P*^b^-value: *P*-values calculated by unconditional logistic regression analysis with adjustment for age, smoking status and alcohol drinking status.**

Supplementary Table S4: The relationship between *IL-16-*rs859*, CYP24A1-*rs4809957 and *FBN1-*rs1042078 and lung squamous cell carcinoma risk.

| Gene | SNP | Model | Genotype | Control  (N = 384) | Case  (N = 98) | Crude Analysis | | Adjusted Analysis | |
| --- | --- | --- | --- | --- | --- | --- | --- | --- | --- |
|  |  |  |  |  |  | OR (95% CI) | *P*^a^-value | OR (95% CI) | *P*^b^-value |
| IL-16  (N = 482) | rs859 (call rate 100%) | Codominant | G/G | 102 (26.6%) | 24 (24.5%) | 1.00 | 0.640 | 1.00 | 0.400 |
|  |  |  | G/A | 190 (49.5%) | 46 (46.9%) | 1.03 (0.59-1.78) |  | 1.06 (0.57-1.97) |  |
|  |  |  | A/A | 92 (24.0%) | 28 (28.6%) | 1.29 (0.70-2.39) |  | 1.55 (0.76-3.14) |  |
|  |  | Dominant | G/G | 102 (26.6%) | 24 (24.5%) | 1.00 | 0.680 | 1.00 | 0.530 |
|  |  |  | G/A-A/A | 282 (73.4%) | 74 (75.5%) | 1.12 (0.67-1.86) |  | 1.20 (0.68-2.14) |  |
|  |  | Recessive | G/G-G/A | 292 (76.0%) | 70 (71.4%) | 1.00 | 0.350 | 1.00 | 0.180 |
|  |  |  | A/A | 92 (24.0%) | 28 (28.6%) | 1.27 (0.77-2.09) |  | 1.49 (0.83-2.67) |  |
|  |  | Log-additive | --- | --- | --- | 1.14 (0.84-1.56) | 0.410 | 1.24 (0.87-1.78) | 0.230 |
|  | | | | | | | | | |
| CYP24A1  (N = 482) | rs4809957 (call rate 100%) | Codominant | G/G | 143 (37.2%) | 32 (32.6%) | 1.00 | 0.560 | 1.00 | 0.950 |
|  |  |  | G/A | 185 (48.2%) | 48 (49.0%) | 1.16 (0.70-1.91) |  | 1.07 (0.61-1.89) |  |
|  |  |  | A/A | 56 (14.6%) | 18 (18.4%) | 1.44 (0.75-2.77) |  | 0.97 (0.45-2.07) |  |
|  |  | Dominant | G/G | 143 (37.2%) | 32 (32.6%) | 1.00 | 0.400 | 1.00 | 0.870 |
|  |  |  | G/A-A/A | 241 (62.8%) | 66 (67.3%) | 1.22 (0.76-1.96) |  | 1.05 (0.61-1.79) |  |
|  |  | Recessive | G/G-G/A | 328 (85.4%) | 80 (81.6%) | 1.00 | 0.360 | 1.00 | 0.830 |
|  |  |  | A/A | 56 (14.6%) | 18 (18.4%) | 1.32 (0.73-2.36) |  | 0.93 (0.47-1.83) |  |
|  |  | Log-additive | --- | --- | --- | 1.19 (0.87-1.64) | 0.280 | 1.00 (0.69-1.44) | 0.990 |
|  | | | | | | | | | |
| FBN1  (N = 482) | rs1042078 (call rate 100%) | Codominant | A/A | 117 (30.5%) | 26 (26.5%) | 1.00 | 0.660 | 1.00 | 0.850 |
|  |  |  | A/G | 185 (48.2%) | 52 (53.1%) | 1.26 (0.75-2.14) |  | 1.19 (0.65-2.18) |  |
|  |  |  | G/G | 82 (21.4%) | 20 (20.4%) | 1.10 (0.57-2.10) |  | 1.13 (0.54-2.38) |  |
|  |  | Dominant | A/A | 117 (30.5%) | 26 (26.5%) | 1.00 | 0.440 | 1.00 | 0.580 |
|  |  |  | A/G-G/G | 267 (69.5%) | 72 (73.5%) | 1.21 (0.74-2.00) |  | 1.18 (0.66-2.09) |  |
|  |  | Recessive | A/A-A/G | 302 (78.7%) | 78 (79.6%) | 1.00 | 0.840 | 1.00 | 0.980 |
|  |  |  | G/G | 82 (21.4%) | 20 (20.4%) | 0.94 (0.55-1.63) |  | 1.01 (0.54-1.89) |  |
|  |  | Log-additive | --- | --- | --- | 1.06 (0.78-1.45) | 0.710 | 1.07 (0.74-1.55) | 0.710 |

**SNP: Single Nucleotide Polymorphism; OR: Odds Ratio; 95% CI: 95% Confidence Interval.**

***P*^a^-value: *P*-values calculated by unconditional logistic regression analysis.**

***P*^b^-value: *P*-values calculated by unconditional logistic regression analysis with adjustment for age, gender, smoking status and alcohol drinking status.**

Supplementary Table S5: The effects of *IL-16-*rs859*, CYP24A1-*rs4809957 and *FBN1-*rs1042078 on TNM staging of lung cancer.

| Gene | SNP | Model | Genotype | I-II Stage  (N = 75) | III-IV Stage  (N = 213) | Crude Analysis | | Adjusted Analysis | |
| --- | --- | --- | --- | --- | --- | --- | --- | --- | --- |
|  |  |  |  |  |  | OR (95% CI) | *P*^a^-value | OR (95% CI) | *P*^b^-value |
| IL-16  (N = 288) | rs859 (call rate 99.65%) | Codominant | A/A | 25 (33.8%) | 51 (23.9%) | 1.00 | 0.260 | 1.00 | 0.210 |
|  |  |  | G/A | 35 (47.3%) | 117 (54.9%) | 1.64 (0.89-3.01) |  | 1.72 (0.93-3.19) |  |
|  |  |  | G/G | 14 (18.9%) | 45 (21.1%) | 1.58 (0.73-3.39) |  | 1.64 (0.76-3.57) |  |
|  |  | Dominant | A/A | 25 (33.8%) | 51 (23.9%) | 1.00 | 0.100 | 1.00 | 0.080 |
|  |  |  | G/A-G/G | 49 (66.2%) | 162 (76.1%) | 1.62 (0.91-2.88) |  | 1.70 (0.95-3.05) |  |
|  |  | Recessive | A/A-G/A | 60 (81.1%) | 168 (78.9%) | 1.00 | 0.680 | 1.00 | 0.670 |
|  |  |  | G/G | 14 (18.9%) | 45 (21.1%) | 1.15 (0.59-2.24) |  | 1.16 (0.59-2.26) |  |
|  |  | Log-additive | --- | --- | --- | 1.30 (0.88-1.92) | 0.190 | 1.33 (0.89-1.97) | 0.160 |
|  | | | | | | | | | |
| CYP24A1  (N = 288) | rs4809957 (call rate 100%) | Codominant | G/G | 18 (24.0%) | 95 (44.6%) | 1.00 | ***0.0057*** | 1.00 | ***0.0067*** |
|  |  |  | G/A | 45 (60.0%) | 93 (43.7%) | **0.39 (0.21-0.73)** |  | **0.40 (0.21-0.74)** |  |
|  |  |  | A/A | 12 (16.0%) | 25 (11.7%) | **0.39 (0.17-0.93)** |  | **0.38 (0.16-0.91)** |  |
|  |  | Dominant | G/G | 18 (24.0%) | 95 (44.6%) | 1.00 | ***0.0013*** | 1.00 | ***0.0016*** |
|  |  |  | G/A-A/A | 57 (76.0%) | 118 (55.4%) | **0.39 (0.22-0.71)** |  | **0.40 (0.22-0.72)** |  |
|  |  | Recessive | G/G-G/A | 63 (84.0%) | 188 (88.3%) | 1.00 | 0.350 | 1.00 | 0.290 |
|  |  |  | A/A | 12 (16.0%) | 25 (11.7%) | 0.70 (0.33-1.47) |  | 0.66 (0.31-1.42) |  |
|  |  | Log-additive | --- | --- | --- | **0.58 (0.39-0.86)** | ***0.0061*** | **0.57 (0.38-0.85)** | ***0.0054*** |
|  | | | | | | | | | |
| FBN1  (N = 288) | rs1042078 (call rate 100%) | Codominant | A/A | 22 (29.3%) | 55 (25.8%) | 1.00 | 0.740 | 1.00 | 0.770 |
|  |  |  | A/G | 43 (57.3%) | 123 (57.8%) | 1.14 (0.63-2.09) |  | 1.13 (0.61-2.07) |  |
|  |  |  | G/G | 10 (13.3%) | 35 (16.4%) | 1.40 (0.59-3.31) |  | 1.37 (0.58-3.25) |  |
|  |  | Dominant | A/A | 22 (29.3%) | 55 (25.8%) | 1.00 | 0.560 | 1.00 | 0.600 |
|  |  |  | A/G-G/G | 53 (70.7%) | 158 (74.2%) | 1.19 (0.66-2.14) |  | 1.17 (0.65-2.11) |  |
|  |  | Recessive | A/A-A/G | 65 (86.7%) | 178 (83.6%) | 1.00 | 0.520 | 1.00 | 0.550 |
|  |  |  | G/G | 10 (13.3%) | 35 (16.4%) | 1.28 (0.60-2.73) |  | 1.26 (0.59-2.70) |  |
|  |  | Log-additive | --- | --- | --- | 1.18 (0.78-1.78) | 0.440 | 1.16 (0.77-1.76) | 0.480 |

**SNP: Single Nucleotide Polymorphism; OR: Odds Ratio; 95% CI: 95% Confidence Interval.**

***P*^a^-value: *P*-values calculated by unconditional logistic regression analysis.**

***P*^b^-value: *P*-values calculated by unconditional logistic regression analysis with adjustment for age, gender, smoking status and alcohol drinking status.**

**Bold italics indicates the statistical significance (*P* < 0.05).**

Supplementary Table S6: The effects of *IL-16-*rs859*, CYP24A1-*rs4809957 and *FBN1-*rs1042078 on lymph node metastasis of lung cancer.

| Gene | SNP | Model | Genotype | Negative Case  (N = 127) | Positive Case  (N = 195) | Crude Analysis | | Adjusted Analysis | |
| --- | --- | --- | --- | --- | --- | --- | --- | --- | --- |
|  |  |  |  |  |  | OR (95% CI) | *P*^a^-value | OR (95% CI) | *P*^b^-value |
| IL-16  (N = 322) | rs859 (call rate 99.69%) | Codominant | A/A | 32 (25.4%) | 57 (29.2%) | 1.00 | 0.750 | 1.00 | 0.770 |
|  |  |  | G/A | 66 (52.4%) | 98 (50.3%) | 0.83 (0.49-1.42) |  | 0.85 (0.50-1.46) |  |
|  |  |  | G/G | 28 (22.2%) | 40 (20.5%) | 0.80 (0.42-1.53) |  | 0.80 (0.41-1.53) |  |
|  |  | Dominant | A/A | 32 (25.4%) | 57 (29.2%) | 1.00 | 0.450 | 1.00 | 0.490 |
|  |  |  | G/A-G/G | 94 (74.6%) | 138 (70.8%) | 0.82 (0.50-1.37) |  | 0.83 (0.50-1.39) |  |
|  |  | Recessive | A/A-G/A | 98 (77.8%) | 155 (79.5%) | 1.00 | 0.710 | 1.00 | 0.670 |
|  |  |  | G/G | 28 (22.2%) | 40 (20.5%) | 0.90 (0.52-1.56) |  | 0.89 (0.51-1.54) |  |
|  |  | Log-additive | --- | --- | --- | 0.89 (0.65-1.23) | 0.490 | 0.89 (0.64-1.23) | 0.490 |
|  | | | | | | | | | |
| CYP24A1  (N = 322) | rs4809957 (call rate 100%) | Codominant | G/G | 40 (31.5%) | 84 (43.1%) | 1.00 | 0.110 | 1.00 | 0.110 |
|  |  |  | G/A | 66 (52.0%) | 86 (44.1%) | 0.62 (0.38-1.02) |  | 0.63 (0.38-1.04) |  |
|  |  |  | A/A | 21 (16.5%) | 25 (12.8%) | 0.57 (0.28-1.13) |  | 0.54 (0.26-1.09) |  |
|  |  | Dominant | G/G | 40 (31.5%) | 84 (43.1%) | 1.00 | ***0.036*** | 1.00 | ***0.039*** |
|  |  |  | G/A-A/A | 87 (68.5%) | 111 (56.9%) | **0.61 (0.38-0.97)** |  | **0.61 (0.38-0.98)** |  |
|  |  | Recessive | G/G-G/A | 106 (83.5%) | 170 (87.2%) | 1.00 | 0.350 | 1.00 | 0.270 |
|  |  |  | A/A | 21 (16.5%) | 25 (12.8%) | 0.74 (0.40-1.39) |  | 0.69 (0.36-1.32) |  |
|  |  | Log-additive | --- | --- | --- | 0.72 (0.52-1.00) | 0.050 | **0.71 (0.51-0.99)** | ***0.042*** |
|  | | | | | | | | | |
| FBN1  (N = 322) | rs1042078 (call rate 100%) | Codominant | A/A | 40 (31.5%) | 45 (23.1%) | 1.00 | 0.200 | 1.00 | 0.240 |
|  |  |  | A/G | 65 (51.2%) | 118 (60.5%) | 1.61 (0.96-2.72) |  | 1.57 (0.93-2.66) |  |
|  |  |  | G/G | 22 (17.3%) | 32 (16.4%) | 1.29 (0.65-2.58) |  | 1.28 (0.64-2.56) |  |
|  |  | Dominant | A/A | 40 (31.5%) | 45 (23.1%) | 1.00 | 0.096 | 1.00 | 0.120 |
|  |  |  | A/G-G/G | 87 (68.5%) | 150 (76.9%) | 1.53 (0.93-2.53) |  | 1.50 (0.90-2.48) |  |
|  |  | Recessive | A/A-A/G | 105 (82.7%) | 163 (83.6%) | 1.00 | 0.830 | 1.00 | 0.850 |
|  |  |  | G/G | 22 (17.3%) | 32 (16.4%) | 0.94 (0.52-1.70) |  | 0.94 (0.52-1.72) |  |
|  |  | Log-additive | --- | --- | --- | 1.18 (0.78-1.78) | 0.310 | 1.18 (0.84-1.68) | 0.340 |

**SNP: Single Nucleotide Polymorphism; OR: Odds Ratio; 95% CI: 95% Confidence Interval.**

***P*^a^-value: *P*-values calculated by unconditional logistic regression analysis.**

***P*^b^-value: *P*-values calculated by unconditional logistic regression analysis with adjustment for age, gender, smoking status and alcohol drinking status.**

**Bold italics indicates the statistical significance (*P* < 0.05).**

Supplementary Table S7: The relationship between *IL-16-*rs859*, CYP24A1-*rs4809957 and *FBN1-*rs1042078 and lung cancer risk among drinkers.

| Gene | SNP | Model | Genotype | Control  (N = 169) | Case  (N = 93) | Crude Analysis | | Adjusted Analysis | |
| --- | --- | --- | --- | --- | --- | --- | --- | --- | --- |
|  |  |  |  |  |  | OR (95% CI) | *P*^a^-value | OR (95% CI) | *P*^b^-value |
| IL-16  (N = 262) | rs859 (call rate 100%) | Codominant | A/A | 45 (26.6%) | 27 (29.0%) | 1.00 | 0.760 | 1.00 | 0.740 |
|  |  |  | G/A | 81 (47.9%) | 46 (49.5%) | 0.95 (0.52-1.72) |  | 0.96 (0.44-2.08) |  |
|  |  |  | G/G | 43 (25.4%) | 20 (21.5%) | 0.78 (0.38-1.58) |  | 0.74 (0.31-1.76) |  |
|  |  | Dominant | A/A | 45 (26.6%) | 27 (29.0%) | 1.00 | 0.680 | 1.00 | 0.710 |
|  |  |  | G/A-G/G | 124 (73.4%) | 66 (71.0%) | 0.89 (0.51-1.56) |  | 0.87 (0.43-1.79) |  |
|  |  | Recessive | A/A-G/A | 126 (74.6%) | 73 (78.5%) | 1.00 | 0.470 | 1.00 | 0.440 |
|  |  |  | G/G | 43 (25.4%) | 20 (21.5%) | 0.80 (0.44-1.47) |  | 0.75 (0.37-1.55) |  |
|  |  | Log-additive | --- | --- | --- | 0.88 (0.62-1.26) | 0.490 | 0.86 (0.55-1.33) | 0.490 |
|  | | | | | | | | | |
| CYP24A1  (N = 262) | rs4809957 (call rate 100%) | Codominant | G/G | 65 (38.5%) | 35 (37.6%) | 1.00 | 0.430 | 1.00 | 0.150 |
|  |  |  | G/A | 79 (46.8%) | 49 (52.7%) | 1.15 (0.67-1.98) |  | 1.10 (0.55-2.20) |  |
|  |  |  | A/A | 25 (14.8%) | 9 (9.7%) | 0.67 (0.28-1.59) |  | 0.41 (0.14-1.20) |  |
|  |  | Dominant | G/G | 65 (38.5%) | 35 (37.6%) | 1.00 | 0.900 | 1.00 | 0.770 |
|  |  |  | G/A-A/A | 104 (61.5%) | 58 (62.4%) | 1.04 (0.61-1.74) |  | 0.91 (0.47-1.75) |  |
|  |  | Recessive | G/G-G/A | 144 (85.2%) | 84 (90.3%) | 1.00 | 0.230 | 1.00 | 0.053 |
|  |  |  | A/A | 25 (14.8%) | 9 (9.7%) | 0.62 (0.28-1.38) |  | 0.39 (0.15-1.04) |  |
|  |  | Log-additive | --- | --- | --- | 0.91 (0.62-1.33) | 0.620 | 0.75 (0.46-1.21) | 0.230 |
|  | | | | | | | | | |
| FBN1  (N = 262) | rs1042078 (call rate 100%) | Codominant | A/A | 54 (31.9%) | 24 (25.8%) | 1.00 | 0.520 | 1.00 | 0.340 |
|  |  |  | A/G | 85 (50.3%) | 53 (57.0%) | 1.40 (0.78-2.53) |  | 1.71 (0.81-3.61) |  |
|  |  |  | G/G | 30 (17.8%) | 16 (17.2%) | 1.20 (0.55-2.60) |  | 1.20 (0.46-3.12) |  |
|  |  | Dominant | A/A | 54 (31.9%) | 24 (25.8%) | 1.00 | 0.290 | 1.00 | 0.220 |
|  |  |  | A/G-G/G | 115 (68.0%) | 69 (74.2%) | 1.35 (0.77-2.38) |  | 1.55 (0.76-3.17) |  |
|  |  | Recessive | A/A-A/G | 139 (82.2%) | 77 (82.8%) | 1.00 | 0.910 | 1.00 | 0.690 |
|  |  |  | G/G | 30 (17.8%) | 16 (17.2%) | 0.96 (0.49-1.88) |  | 0.85 (0.37-1.92) |  |
|  |  | Log-additive | --- | --- | --- | 1.13 (0.78-1.64) | 0.520 | 1.15 (0.72-1.82) | 0.560 |

**SNP: Single Nucleotide Polymorphism; OR: Odds Ratio; 95% CI: 95% Confidence Interval.**

***P*^a^-value: *P*-values calculated by unconditional logistic regression analysis.**

***P*^b^-value: *P*-values calculated by unconditional logistic regression analysis with adjustment for age, gender and smoking status.**

Supplementary Table S8: The relationship between *IL-16-*rs859*, CYP24A1-*rs4809957 and *FBN1-*rs1042078 and lung cancer risk among smokers.

| Gene | SNP | Model | Genotype | Control  (N = 164) | Case  (N = 182) | Crude Analysis | | Adjusted Analysis | |
| --- | --- | --- | --- | --- | --- | --- | --- | --- | --- |
|  |  |  |  |  |  | OR (95% CI) | *P*^a^-value | OR (95% CI) | *P*^b^-value |
| IL-16  (N = 346) | rs859 (call rate 99.71%) | Codominant | A/A | 40 (24.4%) | 56 (30.9%) | 1.00 | 0.360 | 1.00 | 0.190 |
|  |  |  | G/A | 83 (50.6%) | 87 (48.1%) | 0.75 (0.45-1.24) |  | 0.65 (0.35-1.19) |  |
|  |  |  | G/G | 41 (25.0%) | 38 (21.0%) | 0.66 (0.36-1.21) |  | 0.53 (0.26-1.09) |  |
|  |  | Dominant | A/A | 40 (24.4%) | 56 (30.9%) | 1.00 | 0.170 | 1.00 | 0.086 |
|  |  |  | G/A-G/G | 124 (75.6%) | 125 (69.1%) | 0.72 (0.45-1.16) |  | 0.61 (0.34-1.08) |  |
|  |  | Recessive | A/A-G/A | 123 (75.0%) | 143 (79.0%) | 1.00 | 0.380 | 1.00 | 0.250 |
|  |  |  | G/G | 41 (25.0%) | 38 (21.0%) | 0.80 (0.48-1.32) |  | 0.71 (0.39-1.28) |  |
|  |  | Log-additive | --- | --- | --- | 0.81 (0.60-1.09) | 0.170 | 0.73 (0.51-1.04) | 0.079 |
|  | | | | | | | | | |
| CYP24A1  (N = 346) | rs4809957 (call rate 100%) | Codominant | G/G | 63 (38.4%) | 66 (36.3%) | 1.00 | 0.920 | 1.00 | 0.660 |
|  |  |  | G/A | 75 (45.7%) | 86 (47.2%) | 1.09 (0.69-1.74) |  | 0.96 (0.56-1.67) |  |
|  |  |  | A/A | 26 (15.8%) | 30 (16.5%) | 1.10 (0.59-2.06) |  | 0.71 (0.33-1.53) |  |
|  |  | Dominant | G/G | 63 (38.4%) | 66 (36.3%) | 1.00 | 0.680 | 1.00 | 0.680 |
|  |  |  | G/A-A/A | 101 (61.6%) | 116 (63.7%) | 1.10 (0.71-1.70) |  | 0.90 (0.53-1.51) |  |
|  |  | Recessive | G/G-G/A | 138 (84.2%) | 152 (83.5%) | 1.00 | 0.870 | 1.00 | 0.370 |
|  |  |  | A/A | 26 (15.8%) | 30 (16.5%) | 1.05 (0.59-1.86) |  | 0.73 (0.37-1.45) |  |
|  |  | Log-additive | --- | --- | --- | 1.06 (0.78-1.43) | 0.710 | 0.87 (0.60-1.25) | 0.450 |
|  | | | | | | | | | |
| FBN1  (N = 346) | rs1042078 (call rate 100%) | Codominant | A/A | 45 (27.4%) | 45 (24.7%) | 1.00 | 0.170 | 1.00 | 0.310 |
|  |  |  | A/G | 83 (50.6%) | 109 (59.9%) | 1.31 (0.79-2.17) |  | 1.29 (0.71-2.34) |  |
|  |  |  | G/G | 36 (21.9%) | 28 (15.4%) | 0.78 (0.41-1.48) |  | 0.79 (0.37-1.67) |  |
|  |  | Dominant | A/A | 45 (27.4%) | 45 (24.7%) | 1.00 | 0.570 | 1.00 | 0.660 |
|  |  |  | A/G-G/G | 119 (72.6%) | 137 (75.3%) | 1.15 (0.71-1.86) |  | 1.14 (0.64-2.01) |  |
|  |  | Recessive | A/A-A/G | 128 (78.0%) | 154 (84.6%) | 1.00 | 0.120 | 1.00 | 0.200 |
|  |  |  | G/G | 36 (21.9%) | 28 (15.4%) | 0.65 (0.37-1.12) |  | 0.66 (0.35-1.25) |  |
|  |  | Log-additive | --- | --- | --- | 0.92 (0.67-1.26) | 0.590 | 0.91 (0.63-1.33) | 0.640 |

**SNP: Single Nucleotide Polymorphism; OR: Odds Ratio; 95% CI: 95% Confidence Interval.**

***P*^a^-value: *P*-values calculated by unconditional logistic regression analysis.**

***P*^b^-value: *P*-values calculated by unconditional logistic regression analysis with adjustment for age, gender and alcohol drinking status.**

Supplementary Table S9: The relationship between *IL-16-*rs859*, CYP24A1-*rs4809957 and *FBN1-*rs1042078 and lung cancer risk among non-smokers.

| Gene | SNP | Model | Genotype | Control  (N = 220) | Case  (N = 140) | Crude Analysis | | Adjusted Analysis | |
| --- | --- | --- | --- | --- | --- | --- | --- | --- | --- |
|  |  |  |  |  |  | OR (95% CI) | *P*^a^-value | OR (95% CI) | *P*^b^-value |
| IL-16  (N = 360) | rs859 (call rate 100%) | Codominant | G/G | 61 (27.7%) | 30 (21.4%) | 1.00 | 0.360 | 1.00 | 0.190 |
|  |  |  | G/A | 107 (48.6%) | 77 (55.0%) | 1.46 (0.86-2.48) |  | 1.65 (0.95-2.86) |  |
|  |  |  | A/A | 52 (23.6%) | 33 (23.6%) | 1.29 (0.70-2.39) |  | 1.55 (0.81-2.99) |  |
|  |  | Dominant | G/G | 61 (27.7%) | 30 (21.4%) | 1.00 | 0.180 | 1.00 | 0.070 |
|  |  |  | G/A-A/A | 159 (72.3%) | 110 (78.6%) | 1.41 (0.85-2.32) |  | 1.62 (0.96-2.74) |  |
|  |  | Recessive | G/G-G/A | 168 (76.4%) | 107 (76.4%) | 1.00 | 0.990 | 1.00 | 0.710 |
|  |  |  | A/A | 52 (23.6%) | 33 (23.6%) | 1.00 (0.60-1.64) |  | 1.10 (0.65-1.87) |  |
|  |  | Log-additive | --- | --- | --- | 1.14 (0.84-1.54) | 0.410 | 1.25 (0.90-1.73) | 0.180 |
|  | | | | | | | | | |
| CYP24A1  (N = 360) | rs4809957 (call rate 100%) | Codominant | G/G | 80 (36.4%) | 58 (41.4%) | 1.00 | 0.590 | 1.00 | 0.630 |
|  |  |  | G/A | 110 (50.0%) | 66 (47.1%) | 0.83 (0.52-1.30) |  | 0.84 (0.52-1.35) |  |
|  |  |  | A/A | 30 (13.6%) | 16 (11.4%) | 0.74 (0.37-1.47) |  | 0.73 (0.35-1.50) |  |
|  |  | Dominant | G/G | 80 (36.4%) | 58 (41.4%) | 1.00 | 0.340 | 1.00 | 0.370 |
|  |  |  | G/A-A/A | 140 (63.6%) | 82 (58.6%) | 0.81 (0.52-1.25) |  | 0.81 (0.51-1.28) |  |
|  |  | Recessive | G/G-G/A | 190 (86.4%) | 124 (88.6%) | 1.00 | 0.540 | 1.00 | 0.530 |
|  |  |  | A/A | 30 (13.6%) | 16 (11.4%) | 0.82 (0.43-1.56) |  | 0.81 (0.41-1.58) |  |
|  |  | Log-additive | --- | --- | --- | 0.85 (0.62-1.17) | 0.310 | 0.85 (0.61-1.19) | 0.330 |
|  | | | | | | | | | |
| FBN1  (N = 360) | rs1042078 (call rate 100%) | Codominant | A/A | 72 (32.7%) | 40 (28.6%) | 1.00 | 0.480 | 1.00 | 0.540 |
|  |  |  | A/G | 102 (46.4%) | 74 (52.9%) | 1.31 (0.80-2.13) |  | 1.29 (0.77-2.15) |  |
|  |  |  | G/G | 46 (20.9%) | 26 (18.6%) | 1.02 (0.55-1.89) |  | 1.00 (0.52-1.91) |  |
|  |  | Dominant | A/A | 72 (32.7%) | 40 (28.6%) | 1.00 | 0.400 | 1.00 | 0.470 |
|  |  |  | A/G-G/G | 148 (67.3%) | 100 (71.4%) | 1.22 (0.77-1.93) |  | 1.20 (0.74-1.95) |  |
|  |  | Recessive | A/A-A/G | 174 (79.1%) | 114 (81.4%) | 1.00 | 0.590 | 1.00 | 0.580 |
|  |  |  | G/G | 46 (20.9%) | 26 (18.6%) | 0.86 (0.50-1.47) |  | 0.85 (0.49-1.50) |  |
|  |  | Log-additive | --- | --- | --- | 1.04 (0.77-1.40) | 0.810 | 1.03 (0.75-1.41) | 0.870 |

**SNP: Single Nucleotide Polymorphism; OR: Odds Ratio; 95% CI: 95% Confidence Interval.**

***P*^a^-value: *P*-values calculated by unconditional logistic regression analysis.**

***P*^b^-value: *P*-values calculated by unconditional logistic regression analysis with adjustment for age, gender and alcohol drinking status.**
